# Nutritional, Chemical, and Functional Properties of Wholegrain Einkorn Pasta Through Cooking and Digestion: A Comparative Study with Wholegrain Durum Wheat Pasta

**DOI:** 10.3390/foods14030370

**Published:** 2025-01-23

**Authors:** Dario Mercatante, Mattia Santoni, Lorenzo Nissen, Spyros Didos, Giulia Salvatori, Gianni Jan D’Ambrosio, Alice Farneti, Elena Chiarello, Flavia Casciano, Gianfranco Picone, Evangelia Mouchtaropoulou, Alessandra Bordoni, Francesca Danesi, Anagnostis Argiriou, Georgia Ayfantopoulou, Andrea Gianotti, Maria Teresa Rodriguez-Estrada

**Affiliations:** 1 Department of Agricultural and Food Sciences (DISTAL), University of Bologna, Viale G. Fanin 40, 40127 Bologna, Italy; dario.mercatante2@unibo.it (D.M.); mattia.santoni@unibo.it (M.S.); lorenzo.nissen@unibo.it (L.N.); giulia.salvatori5@unibo.it (G.S.); gianfranco.picone@unibo.it (G.P.); alessandra.bordoni@unibo.it (A.B.); andrea.gianotti@unibo.it (A.G.); maria.rodriguez@unibo.it (M.T.R.-E.); 2 Interdepartmental Centre of Agrifood Industry Research (CIRI Agrifood), University of Bologna, Via Quinto Bucci 336, 47521 Cesena, Italy; 3 Department of Food Science and Nutrition, University of the Aegean (UOA-FNS), University Hill, 81100 Mytilene, Greece; sdidos@certh.gr (S.D.); eva.mouchtaropoulou@certh.gr (E.M.); argiriou@certh.gr (A.A.); 4 Scientific High School “Augusto Righi”, Piazza Aldo Moro 20, 47521 Cesena, Italy; alice.farneti@liceorighi.it; 5 Centre for Research and Technology Hellas, Hellenic Institute of Transport (CERTH/HIT), 6th km Charilaou, Thermi Rd., Thermi, 57001 Thessaloniki, Greece; gea@certh.gr

**Keywords:** antioxidant capacity, bioaccessibility, bioactive compounds, cooking, in vitro digestion, phenolic compounds, phytosterols, prebiotic activity, wholegrain pasta

## Abstract

Despite growing interest in ancient wheat varieties, the functional and nutritional properties of einkorn (*Triticum monococcum*) in cereal-based foods remain not fully elucidated. This study examined the chemical composition and functional properties of wholegrain einkorn pasta through cooking and simulated gastrointestinal digestion, comparing it with conventional *Triticum durum* wheat pasta. While sharing similar macronutrient profiles, einkorn pasta demonstrated higher retention of key compounds including phenolics, tocopherols, and phytosterols throughout cooking and in vitro digestion. Notable findings include enhanced prebiotic activity specifically targeting bifidobacteria populations and preserved antioxidant capacity despite thermal processing. These results demonstrated einkorn’s potential as a functional food ingredient, suggesting its capacity to deliver enhanced nutritional benefits through its unique matrix properties. Our findings provide mechanistic insights into ancient grain functionality in modern food applications, with implications for developing nutritionally enhanced pasta products.

## 1. Introduction

Pasta represents a cornerstone of global food consumption, traditionally manufactured from durum wheat (*Triticum durum*) [1]. Recent years have witnessed increasing attention towards ancient wheat varieties, driven by their enhanced nutritional profiles and potential health advantages compared to modern wheat cultivars [2,3]. This shift reflects growing consumer interest in traditional grains and their potential contributions to both human health and sustainable agriculture.

Among ancient wheat species, einkorn (*Triticum monococcum*) and emmer (*Triticum dicoccum*) have demonstrated superior nutritional characteristics compared to contemporary wheat varieties, including elevated protein content and increased concentrations of bioactive compounds [4,5]. Einkorn, in particular, has garnered attention for its unique protein composition, high carotenoid content, and enhanced antioxidant capacity [6,7,8,9,10,11,12].

While einkorn remains primarily a niche market in Mediterranean countries, growing consumer interest in ancient grains indicates expansion potential [3,13]. Einkorn’s agricultural characteristics offer both challenges and opportunities for sustainable food production. Despite displaying 30–50% lower yields than modern wheat varieties [14], einkorn offers environmental advantages through reduced input requirements in organic farming systems [15,16]. Einkorn’s adaptability to marginal conditions and natural disease resistance [17] contributes to biodiversity conservation and food system resilience while supporting sustainable agricultural practices and fair pricing mechanisms and also fostering economic stability in farming communities [18].

Wholegrain wheat varieties contain various functional components that may contribute to their potential health effects, including prebiotic compounds such as fructans, arabinoxylans, resistant starch, β-glucans, and fructooligosaccharides (FOS) [19]. These components can beneficially modulate gut microbiota composition and function, with distinct profiles observed between modern and ancient wheat varieties [20,21].

The nutritional and functional properties of wheat-based products depend not only on raw materials [22,23] but also on processing conditions—variables that both affect digestibility. Bioaccessibility (defined as the amounts of compounds released from the food matrix in the gastrointestinal tract) is greatly influenced by the food matrix structure and its behavior during gastrointestinal digestion [24,25]. The physicochemical properties of the food matrix, presence of other components that can improve or antagonize accessibility, and food processing technologies all influence bioaccessibility [25,26,27,28]. Processing steps, particularly milling and cooking, can significantly modify the food matrix, affecting the release and absorption of beneficial compounds [29]. Understanding these dynamics through physiologically relevant approaches, such as simulated digestion models that provide standardized screening conditions, is essential for assessing the nutritional value of cereal-based foods [25]. While in vitro models cannot fully replicate human digestion complexity, they enable systematic comparison of food matrices under controlled conditions. Moreover, standard chemical extraction methods often fail to account for matrix-bound compounds and may not accurately reflect physiological conditions in vivo [30].

Despite the growing interest in ancient wheat-based products, systematic comparisons of nutritional and bioactive compound bioaccessibility between einkorn and durum wheat pasta remain limited, especially regarding chromatographic analysis through processing and digestion. Nutrient and bioactive compound bioaccessibility can vary significantly between wheat varieties due to intrinsic properties and processing conditions [31]. Furthermore, there is a lack of studies considering how thermal processing (particularly pasta making and cooking) influence the bioactive-ingredient properties of ancient wheat-based products (such as phenolics, sterols, and tocopherols) and their subsequent bioaccessibility during gastrointestinal digestion.

This research compared wholegrain einkorn and durum wheat pasta, examining their nutritional and chemical composition before and after cooking. We investigated how these components became bioaccessible during digestion, along with their prebiotic potential and antioxidant properties. Through this comprehensive analysis, we aimed at understanding einkorn’s value as a functional food ingredient and its potential contribution to healthier pasta products.

## 2. Materials and Methods

### 2.1. Materials

Chemical reagents including analytical-grade solvents (methanol, chloroform, hexane, ethyl acetate, diethyl ether, pyridine, acetone, *n*-hexane, hydrochloric acid, sodium hydroxide, and perchloric acid), derivatization reagents (sodium methoxide, boron-trifluoride, hexamethyldisilazane, and trimethylchlorosilane), in vitro digestion assay components (α-amylase, pepsin, pancreatin, bile extract, and Pefabloc^®^ SC), analytical standards (fatty acid methyl esters, sterol standards, tocopherol standards, and phenolic acid standards), internal standards (5α-cholestane, tridecanoic acid methyl ester, and betulinol), spectrophotometric reagents (*o*-phthalaldehyde, sodium 2-mercaptoethanesulfonate, Triton X-100, L-glutamic acid, DPPH, and Trolox), and other basic laboratory chemicals (sodium chloride, potassium chloride, di-sodium tetraborate decahydrate, sodium sulfate, and glycerol) were purchased from Sigma-Aldrich (St. Louis, MO, USA). Amyloglucosidase, an amyloglucosidase activity assay kit, and a ᴅ-glucose assay kit were obtained from Megazyme Ltd. (Bray, Ireland).

### 2.2. Sample Description

The analyses were conducted on pasta samples in the typical Italian ’penne’ shape format. Wholegrain einkorn pasta (EP) samples were produced by Prometeo (Urbino, Italy) using stone-ground wholegrain einkorn semolina, which preserves the natural integration of bran and germ components [32,33]. Commercial wholegrain durum wheat pasta (DP) was purchased in a local supermarket (Coop, Bologna, Italy); the wholegrain durum wheat semolina is obtained by firstly separating the bran during industrial milling and then reincorporating it [32,33]. The bran content in wholegrain durum wheat pasta typically aligns with the natural proportion found in the wheat kernel, which is approximately 20–25% of the total grain weight [34], but it can vary from 5% to 30% based on the specific product formulation and manufacturing processes used to meet commercial product specifications [35,36].

### 2.3. Proximate and Elemental Analyses

Proximate and elemental composition analyses were conducted by an accredited external laboratory (Vegezio s.r.l., Cesena, Italy) following standardized methods and regulatory guidelines. All analyses were performed in accordance with official Association of Official Agricultural Chemists (AOAC) methods [37] and European Union Regulation 1169/2011 [38]. Macronutrient content was determined using the following procedures: moisture and total fat content were analyzed using AOAC-approved gravimetric methods [37]. Protein content was determined using the Kjeldahl method following standard AOAC protocols [37]. Total carbohydrate content was calculated by difference, subtracting the sum of protein, total fat, moisture, and ash from 100 g of sample. Total sugars were quantified using the Luff–Schoorl method [39], while dietary fiber content was determined using the enzymatic–gravimetric method according to ISTISAN protocols [40].

Elemental analysis included iron, zinc, and sodium determinations by atomic absorption spectrometry, following ISTISAN methodologies [40]. Salt content was calculated by applying a conversion factor of 2.5 to the sodium concentration, as prescribed by EU Regulation 1169/2011 [38]. The total energy value was calculated as the sum of the energy contributions from individual macronutrients according to the conversion factors specified in the same regulation.

### 2.4. Pasta Cooking Conditions

Pasta cooking conditions were determined according to the American Association of Cereal Chemists (AACC) Method 66-50 [41], related to Pasta and Noodle Cooking Quality-Firmness. Distilled water (300 mL) was placed into a 500 mL beaker and put on a hotplate until it reached a rolling boil. Subsequently, 25 g of each pasta sample were added to the boiling water. The water-to-sample ratio was maintained at a minimum of 10:1 throughout the cooking process. Cooking trials were conducted to determine the appropriate cooking time. Pasta samples were then cooked according to the abovementioned method, and after 10 min, at 30 s intervals, a piece of pasta was removed from the cooking water and squeezed between two pieces of clear plastic. The desired cooking time was recorded when the raw center core of pasta pieces disappeared. After the cooking trials, it was decided to use a 13 min cooking time for EP and 11 min for DP.

### 2.5. In Vitro Simulated Gastrointestinal Digestion

Simulated gastrointestinal digestion was performed following the standardized static INFOGEST protocol [42]. Seven grams of cooked pasta were briefly minced using a domestic food grinder. The digestion process was conducted in a shaking thermostatic bath (WB-MF; FALC, Treviglio, Italy) at 37 °C and consisted of three sequential phases: (i) oral phase—addition of simulated salivary fluid (containing 750 U/mL amylase) at pH 7 for 2 min; (ii) gastric phase—120 min digestion with simulated gastric juice (containing 2000 U/mL pepsin) at pH 3; and (iii) duodenal phase—120 min digestion with simulated pancreatic juice (containing 10 mM bile and 100 U/mL pancreatin) at pH 7.

The digestion process was stopped by adding 50 μL of 100 mM Pefabloc^®^ SC (76307; Sigma-Aldrich, St. Louis, MO, USA) per milliliter of intestinal digesta (5 mM final concentration) to inhibit serine proteases (trypsin and chymotrypsin) [42]. Samples were briefly vortexed and placed on ice. The in vitro digestion experiment was conducted in triplicate for both EP and DP samples, and a blank of digestion using water instead of a food sample was prepared in parallel to evaluate the contribution of the digestion reagents to the measurements.

For protein, lipid, fatty acid, tocopherol, and phenolic analyses, the post-duodenal digesta was processed through high-speed centrifugation at 50,000× *g* for 20 min under refrigerated conditions (4 °C). The supernatant fractions (representing the soluble, bioaccessible fraction) were carefully collected and stored at −80 °C until further analysis. All analyses were performed within 1 week of sample processing to ensure data accuracy and minimize potential degradation of nutrients and bioactive compounds.

#### 2.5.1. Starch Bioaccessibility and Kinetic Modelling

Following the simulated gastrointestinal digestion, starch bioaccessibility was assessed by measuring glucose release after amyloglucosidase (AMG) treatment, to mimic the action of brush border membrane (BBM) enzymes. Digesta samples were first centrifuged at 4500× *g* for 10 min at 4 °C, and eight aliquots of 1 mL were collected. AMG from *Aspergillus niger* (EC 3.2.1.3; Megazyme Ltd., Bray, Ireland), with activity verified using the R-AMGR3 kit (Megazyme Ltd., Bray, Ireland), was added to each sample in a final activity of 30 U/mL (initial activity 220 U/mL in 50% (*v*/*v*) glycerol), following modifications of previously published protocols [43,44,45,46,47]. Baseline samples (T0) received an equivalent volume of water instead of enzyme.

All samples were incubated at 37 °C in a water bath. Samples were collected at specific time points (T30, T60, T90, and T120 min) during the AMG treatment. The enzymatic reaction was terminated at each designated time point by heating the samples at 100 °C for 5 min in a thermoblock, followed by immediate cooling on ice. The samples were then centrifuged (10,000× *g*, 5 min) to obtain clear supernatants.

ᴅ-glucose concentration in the supernatants was determined using a ᴅ-Glucose Assay Kit (K-GLUC, Megazyme Ltd., Bray, Ireland) based on the glucose oxidase–peroxidase method [48]. For each measurement, 100 µL of sample was mixed with 3 mL of glucose oxidase/peroxidase (GOPOD) reagent and incubated at 45 °C for 20 min. Absorbance was measured at 510 nm using a DU^®^ UV/Vis spectrophotometer (Beckman & Coulter, Brea, CA, USA).

Glucose concentrations were calculated by dividing absorbance readings by the glucose standard value (100 µg/100 µL). The results were then converted to milligrams and adjusted for the total digestion volume (56 mL). Values were normalized to the initial food sample weight (7 g) and expressed as grams of glucose per 100 g of food. Final concentrations were adjusted to account for the weight increase during cooking, as the cooking process doubled the initial sample weight.

Starch concentration was calculated from the free ᴅ-glucose concentration using the following formula adapted from Khrisanapant et al. (2021) [49]:

(1) $$Starch\;concentration\;\left(C\right)=Free\;ᴅ\text{-}Glucose\times 0.9$$

This calculation accounts for the conversion of glucose units to ᴅ-glucose units in starch (162/180 = 0.9).

Starch digestion over time was analyzed using a first-order kinetic equation, commonly applied in starch hydrolysis kinetics studies [50,51,52]. The equation used was as follows:

(2) $$C_{t}=C_{\infty }\times \left(1-e^{\left(-kt\right)}\right)$$

where C_t_ is the concentration of digested starch at time t, C_∞_ is the maximum attainable concentration, and k is the first-order rate constant. This model represents starch digestion as an exponential process, consistent with observations from both in vitro and in vivo carbohydrate digestion studies [53,54]. C_∞_ and k parameters were optimized using Excel’s Solver tool by minimizing the sum of squared residuals, following methods described by Englyst et al. (1992) [55].

#### 2.5.2. Protein Bioaccessibility

The degree of protein hydrolysis (DH) was determined using the *o*-phthalaldehyde (OPA) method according to Kopf-Bolanz et al. (2012) [56]. The analysis was performed on both digested samples and undigested cooked samples to quantify free amino groups.

Complete protein breakdown in raw samples was achieved through acid hydrolysis following the Rutherfurd and Gilani [57] protocol. Briefly, samples were ground in liquid nitrogen to a fine powder, and protein aliquots (20 mg, equivalent to approximately 180 mg undigested cooked sample) were put in a Pyrex^®^ glass tube (100 × 18 mm) with a screw cap, and 6 N hydrochloric acid (5 mL) was added. Samples were sparged with nitrogen gas to remove oxygen from the hydrolysis tube; tubes were sealed and incubated at 110 °C in an ECOCELL^®^-55 oven (MMM Medcenter Munich, Germany). After 24 h, samples were removed from the oven, allowed to cool to room temperature, and then vortexed and centrifuged (3 min at 2600× *g*). The hydrolysates were dried under nitrogen stream using an insufflation device (MPM Instruments; Bernareggio, Italy) and were reconstituted in 1 mL deionized water with vortex mixing. Hydrolyzates were filtered through a 0.22 μm membrane syringe filter to remove insoluble material and particulates. Processed samples were stored at 4 °C and analyzed within 72 h.

Digested samples (1 mL) were mixed with a methanol/water solution (4:1, *v*/*v*; 1 mL) for protein precipitation [58,59]. Following incubation (−20 °C, 1 h) and centrifugation at 2000× *g* (4 °C, 10 min) to remove precipitated protein, supernatants were collected for analysis.

The OPA working solution was freshly prepared by combining 12.5 mL of 0.1 M di-sodium tetraborate decahydrate, 2.5 mL of 10% (*w*/*w*) sodium dodecyl sulfate (SDS), 0.5 mL of 40 g/L OPA in ethanol (approximately 0.3 M), 0.5 mL of 200 g/L sodium 2-mercaptoethanesulfonate (Na-MES; approximately 1.2 M), and 1.25 mL of 100 g/L Triton X-100, with distilled water added to a final volume of 25 mL. The solution was kept in a dark bottle due to light sensitivity. For analysis, OPA solution (232 μL) was combined with 8 μL of sample (hydrolyzed or digested) in a 96-well UV-transparent plate (Greiner UV-Star^®^ Microplate; Kremsmünster, Austria) and thoroughly mixed. After dark incubation (30 °C, 10 min) in a StableTemp Dry Block Heater (Cole-Parmer; Vernon Hills, IL, USA), absorbance was measured at 335 nm using a Tecan M200 spectrophotometer (Männedorf, Switzerland). Primary amino groups were quantified using a L-glutamic acid standard curve (0–8 mM) prepared in 0.5 M perchloric acid.

The protein hydrolysis degree was calculated according to the following equation, adapted from Pineda-Vadillo et al. (2020) [60]:

(3) $$Protein\;DH\left(\%\right)=\left(\frac{mmol\;free\;NH_{2}}{mmol\;total\;NH_{2}}\right)\times 100$$

where free NH_2_ represents the primary amino group content in digested samples (8 µL analyzed; equivalent to 0.5 mg cooked sample), and total NH_2_ represents the primary amino group content in acid-hydrolyzed undigested samples (8 µL analyzed; equivalent to 1.44 mg cooked sample). Values were proportionally normalized to calculate the DH based on equivalent amounts of the initial sample.

### 2.6. Lipid Extraction

Lipids from pasta (raw and cooked), before and after in vitro digestion, were extracted according to a modified version of the Folch method [61]. About 25 g of samples were subjected to extraction by using a chloroform–methanol solution (1:1, *v*/*v*), followed by the addition of another 100 mL of chloroform. Afterwards, 1 M KCl was added, allowing the organic phase to separate. The solution was then taken to dryness, and the fat content was determined gravimetrically.

### 2.7. Total Lipid Profile

Gas chromatography–flame ionization detection (GC-FID) was used to determine the qualitative–quantitative profile of the main lipid classes (free fatty acids, FFA; monoacylglycerols, MAG; free sterols, STE; diacylglycerols, DAG; esterified sterols, E-STE; triacylglycerols, TAG) of pasta (raw and cooked), before and after in vitro digestion.

The methodology described by Gallina Toschi et al. (2014) [62] was followed. About 20 mg of extracted lipids were added with 1 mL of *n*-hexane and placed in a vial with a screw cap; 1 µL was then injected into a GC-FID (Shimadzu, Kyoto, Japan). The different lipid classes (FFA, STE, E-STE, MAG, DAG, and TAG) were identified using various commercial standards. The amount of each lipid class was determined using the internal standard method, with 5α-cholestane as the internal standard (added during the lipid extraction process) and the response factor of each major lipid class (estimated using commercial standards) [63]. The results were expressed as g/100 g of fat.

### 2.8. Total Fatty Acids Profile

To determine the fatty acid composition of pasta (raw and cooked), before and after in vitro digestion, a double methylation in methanolic medium was carried out, first with sodium methoxide and then with boron-trifluoride, to ensure that all fatty acids (FA) (including the free ones) were completely methylated [64].

About 20 mg of the lipid fraction were weighed and added with tridecanoic acid methyl ester (C13:0) as the internal standard. After the double methylation, 1 μL of the phase containing the analytes was injected into a GC-FID (Finson instrument, Glasgow, UK), under the same analytical condition as Cardenia et al. (2015) [65]. Each fatty acid was identified by comparing its retention time with that of a commercial fatty acid methyl ester standard solution. The GC response factor of each fatty acid was calculated by using the FAME standard mix and the internal standard (C13:0). The quantification of FAME was carried out according to the internal standard method. The results were expressed as g/100 g of FAME.

### 2.9. Sterols Profile

To determine the total sterols composition of pasta (raw and cooked), before and after in vitro digestion, the cold saponification process and extraction method described by Sander et al. (1989) [66] was followed.

Approximately 250 mg of lipids and 5 mL of the bioaccessible fraction were weighed, added with betulinol as the internal standard, and cold saponified. Extracted sterols were then derivatized with a silylating mixture (pyridine–hexamethyldisilazane–trimethylchlorosilane, 5:2:1, *v*/*v*/*v*), and injected into a GC coupled with a mass spectrometer (GC-MS, Shimadzu, Kyoto, Japan), using the analytical conditions described by Blanco Morales et al. (2024) [67]. The acquisition and integration modes were, respectively, total ion current (TIC) and single ion monitoring (SIM). The sterols were identified and quantified through characteristic ions using calibration curves constructed with sterol standard solutions, as reported in Blanco Morales et al. (2024) [67]. The results were expressed as g/100 g of sample.

### 2.10. Tocopherols

Tocopherols were quantified in the lipids extracted from pasta (raw and cooked), before and after in vitro digestion, according to the method of Tosi et al. (2004) [68].

About 10 mg of lipids were cold saponified; the unsaponifiable fraction was extracted and injected into a HPLC coupled to a fluorimeter (HPLC-FD; Agilent Technologies, Santa Clara, CA, USA). The HPLC instrument used was an HP 1050 series system (Hewlett-Packard, Palo Alto, CA, USA), equipped with a 20 µL loop injector (Rheodyne, Cotati, CA, USA), a 10 µm Luna silica column (250 × 4.6 mm internal diameter; Phenomenex, Torrance, CA, USA), and an HP 1100 series fluorimeter (Hewlett-Packard, Palo Alto, CA, USA). Chromatographic analyses were conducted under isocratic conditions with a mobile phase consisting of *n*-hexane–ethylacetate–acetic acid (97.3:1.8:0.9, *v*/*v*/*v*) at a flow rate of 1.6 mL/minute.

The fluorescence measurements were performed at 290/330 nm (excitation/emission). Tocopherols were quantified using five-point calibration curves (0.5–50 μg/mL, R^2^ > 0.99) constructed with α- and β-tocopherol standards (Sigma-Aldrich, St. Louis, MO, USA). The results were expressed as mg/kg of sample.

### 2.11. Phenolic Acids Composition

The extraction of phenolic acids from pasta (raw and cooked), before and after in vitro digestion, was carried out according to Cardenia et al. (2018) [69].

For the extraction of soluble conjugated phenolic acids, exactly 0.5 g of each pasta samples were mixed with a methanol–acetone–water (7:7:6, *v*/*v*/*v*) solution, digested with an alkaline hydrolyzing solution (4 M NaOH), and acidified using 6 M HCl to achieve a pH value of about 2. The resulting mixture was extracted with diethyl ether–ethyl acetate (1:1, *v*/*v*). After centrifugation, the extracts were clarified with sodium sulfate, decanted into a clean flask, and evaporated to dryness. The dry extract was reconstituted with a methanol–water (1:1, *v*/*v*) solution, filtered and injected into a high-performance liquid chromatography system coupled to a diode array detector (HPLC-DAD) (Agilent Technologies, Santa Clara, CA, USA).

For the extraction of insoluble bound phenolic acids, after removing the supernatant obtained with the hydroalcoholic extraction of soluble conjugated phenolic acids, the remaining pellet was also subjected to alkaline hydrolysis followed by acidification, extraction, centrifugation, clarification, decantation, and evaporation to dryness, using the same conditions as previously described [69]. The dry extract was reconstituted with a methanol–water (1:1, *v*/*v*) solution, filtered, and injected into the HPLC-DAD (Agilent Technologies, Santa Clara, CA, USA).

An HPLC system (LC series 1260, Agilent Technologies, Palo Alto, CA, USA) equipped with a 1100 series autosampler, a 1050 series Diode Array Detector (DAD), and an analytical SphereClone ODS (2) column (5 µm particle size; 250 × 4.60 mm internal diameter; Phenomenex, Torrance, CA, USA) was employed. A 20 µL aliquot of each sample was injected into the HPLC system, operating at a flow rate of 1.5 mL/minute. The column temperature was maintained at 30 ± 1 °C. Elution was carried out using a binary solvent system: solvent A (1% *v*/*v* acetic acid in water) and solvent B (methanol). A multi-segment gradient was applied with the following program: 15% solvent B at the start of the run, 20% solvent B at 10 min, 35% solvent B from 16 to 28 min, and 15% solvent B from 30 to 34 min.

Phenolic acids were detected at 280 nm; they were identified by comparing their retention times and spectra with those of pure standards and spiking samples with standard solutions. Phenolic acids were quantified using five-point calibration curves (R^2^ > 0.99) constructed with commercial standards (Sigma-Aldrich, St. Louis, MO, USA): gallic acid (2.45–1054 μg/mL), ferulic acid (2.33–1040 μg/mL), *p*-coumaric acid (1.28–25.00 μg/mL), caffeic acid (0.88–17.15 μg/mL), syringic acid (0.78–15.20 μg/mL), and chlorogenic acid (1.18–28.00 μg/mL). The results were expressed as mg/kg of sample.

### 2.12. Total Antioxidant Capacity

The extraction protocol for free and bound antioxidant compounds was adapted from previously published methods [70,71] with slight adjustments. Ground pasta samples (0.5 g) underwent free phenolic extraction with 80% methanol (5 mL) using homogenization and sonication in an ultrasonic bath (Branson Ultrasonics, Danbury, CT, USA). After centrifugation (2500× *g*, 10 min), the supernatant was collected, and the extraction was repeated twice on the residue with fresh methanol (5 mL, 80%). All supernatants were combined and dried at 45 °C using a rotary evaporator under vacuum (free phenolic compounds). The remaining residue was then digested with 10 mL of 2 M NaOH at room temperature for 2 h. The pH of the solution was adjusted to 2, followed by centrifugation at 2500× *g* for 5 min. The resulting supernatant was extracted three times using a 1:1 (*v*/*v*) mixture of ethyl acetate–diethyl ether. The collected organic layers were evaporated to dryness (bound antioxidant compounds). The dried residues containing free and bound antioxidant compounds were re-dissolved in 2 mL of 80% methanol, filtered through a 0.45 μm filter, and the resulting extracts were used for determining for total antioxidant activity (TAC) assays.

TAC was evaluated only for raw and cooked pasta samples, since bile salts and digestive enzymes used during in vitro digestion can act as antioxidants themselves, interfering with the measurements and leading to overestimated results [72,73,74,75].

#### 2.12.1. DPPH Assay

Antioxidant activity was assessed using the 2,2′-diphenyl-1-picrylhydrazyl (DPPH) radical scavenging method [76]. Extract samples (25 μL) were combined with freshly prepared DPPH solution (975 μL, 6 × 10^−9^ mol/L) and incubated at room temperature for 30 min. Absorbance measurements were performed at 515 nm using a spectrophotometer UV-2600i (Shimadzu, Kyoto, Japan), with results expressed as percentage DPPH neutralization.

#### 2.12.2. FRAP Assay

Ferric reducing antioxidant power (FRAP) was determined following established protocols [77]. The assay involved mixing extract (1 mL) with methanolic FRAP solution (3 mL) and measuring absorbance after 10 min at 593 nm against a water blank. Results were expressed as μmol Trolox equivalents per 100 g dry weight (μmol TE/100 g).

### 2.13. Microbiological Analysis

All probiotic microbial strains were obtained from commercial cultures (Bromatech, Albese, CO, Italy) or deposited type strains or probiotic-like strains belonging to the Culture Collection of DISTAL at the University of Bologna. *Escherichia coli* strains were deposited type strains. Probiotics have been previously isolated from commercial supplements and repeatedly propagated in our laboratory [78]. *Lactiplantibacillus plantarum subsp. plantarum* NCIMB 8299, *Lactobacillus acidophilus* LA1, *Lactobacillus rhamnosus C1112 Lactobacillus rhamnosus* HN001, *Lactobacillus reuterii* LR92, *Bifidobacterium bifidum NCIMB 700795, Bifidobacterium lactis* BL-04, *Bifidobacterium bifidum* BB-06, *Bifidobacterium breve* BB-03, *Bifidobacterium longum* BL-05, *E. coli* ATCC 25922, and *E. coli* NCIMB 555 were cultured from glycerol stocks stored at −80 °C and were propagated in selective media (Oxoid, Thermo Fisher Scientific, Waltham, MA, USA) at specific conditions [79].

### 2.14. Bacterial Culture-Dependent and Independent Quantifications

For all bacteria, 1 mL of each sample was aseptically transferred into a sterile tube with 9 mL of physiological solution (0.9 g/dL NaCl) to be serially diluted (1/10) and plated. The lactobacilli mix was counted on MRS (Man, Rogosa, and Sharpe) agar (Oxoid, Thermo Fisher Scientific, Waltham, MA, USA) after propagation for at least 24 h at 37 °C in jars with anaerobiosis catalyst (Oxoid, Thermo Fischer Scientific, Waltham, MA, USA). The bifidobacteria mix was counted on MRS agar supplemented with 0.05 g/dL L-cysteine after propagation in the same conditions of the lactobacilli. The *E. coli* mix was counted on BHI (brain heart infusion) agar (Oxoid, Thermo Fisher Scientific, Waltham, MA, USA) after propagation at 37 °C for 24 h.

Bacterial DNA from a prebiotic activity assay was extracted with the Pure Link Microbiome kit (Invitrogen, Thermo Fisher Scientific, Waltham, MA, USA). Genetic standards for quantitative polymerase chain reaction (qPCR) were prepared from serially diluted PCR products (1/10), and amplified gene targets were obtained with specific primers (Table S1) with the ProFlex PCR System (Thermo Fisher Scientific, Waltham, MA, USA) and SuperFi Platinum Taq (Thermo Fisher Scientific, Waltham, MA, USA) and were purified with a GeneJet PCR purification kit (Thermo Fisher Scientific, Waltham, MA, USA). qPCR was performed with a QuantStudio 5 (Applied Biosystem, Waltham, MA, USA) and QuantStudio Design and Analyse 2.1 (Applied Biosystem, Waltham, MA, USA) software. PCR and qPCR reactions were performed according to previously published protocols [80].

### 2.15. Prebiotic Activity

Cooked pasta samples were used for estimating the prebiotic score, as previously described [81,82], including qPCR quantifications [79]. Cooked pasta samples were freeze dried using a Savant freeze-dryer Lyolab 3000 apparatus (Thermo Fisher Scientific, Waltham, MA, USA), in order to add 1 g/dL of product to 10 mL of culture media. FOS from chicory was used as a prebiotic positive control, and 1 g/dL of glucose was used as a negative control. All bacterial mixtures were used at the final concentration of 6 Log_10_ CFU/mL [82]. The prebiotic potential was obtained by statistical comparison of the results obtained from the microbial analyses and the metabolic analyses and also some nutritional data, such as the content of protein, carbohydrates, or fibers. This study was conducted employing normalized datasets of both types of significant variables (microbial and nutritional).

### 2.16. Statistical Analysis

Each experiment was conducted with three independent replicates, with chemical extractions and subsequent analytical measurements performed in triplicate, except for AMG treatment sampling and bacterial plating which were run in duplicates. Results are expressed as mean values with standard deviation (SD). Data normality (*p* < 0.05) was evaluated using the Shapiro–Wilk test, while variance homogeneity (*p* < 0.05) was assessed using both Levene and Bartlett tests. Chemical and TAC data underwent two-way analysis of variance (ANOVA), with type, cooking, and digestion as primary factors, including their interactions (Type × Cooking; Type × Digestion). Mean differentiation employed Tukey’s honest significance test at the 95% confidence level (*p* < 0.05). All chemical analyses were performed using XL-STAT software (version 7.5.2, Addinsoft, Paris, France). Prebiotic activity data were first assessed for normality (Shapiro–Wilk test) and homoscedasticity (Levene’s test), followed by ANOVA and Tukey’s post hoc test (*p* < 0.05). Visualization of the enumeration of microbes by qPCR and the prebiotic scores utilized boxplots in the R environment. Correlation analysis employed Spearman rank methodology in the R environment, generating two-way joining heatmaps with dendrogram hierarchy [83]. Statistical differences in proximate composition, starch, and protein bioaccessibility were analyzed using an unpaired *t*-test with GraphPad Prism version 10.3 (GraphPad Software, Inc., San Diego, CA, USA).

## 3. Results

### 3.1. Proximate and Elemental Analyses

No significant differences were observed between EP and DP samples across all nutritional parameters (Table 1; *p* > 0.05 for all comparisons, n = 3). Both products contained approximately 1500 kJ per 100 g (approximately 1200 kJ per 80 g portion), with carbohydrates as the main energy source. The fiber content exceeded 6 g/100 g in both pasta samples.

### 3.2. Starch Bioaccessibility

**Figure 1:**
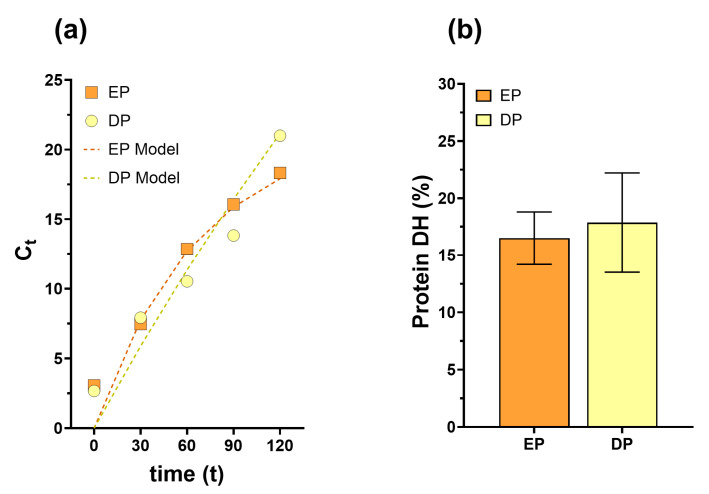
Starch and protein bioaccessibility. (**a**) Starch digestion kinetics of EP and DP over 120 min of in vitro digestion with amyloglucosidase. Data points show experimental measurements of digested starch concentration (C_t_) at different time points (0, 30, 60, 90, and 120 min), while dashed lines represent the fitted first-order kinetic model curves. (**b**) Degree of protein hydrolysis (DH%) in EP and DP after in vitro digestion, as determined by the *o*-phthalaldehyde (OPA) method. DH% represents the percentage of free amino groups relative to total amino nitrogen content. Statistical analysis using unpaired *t*-test revealed no significant differences between the two pasta types (*p* = 0.7949).

The starch digestion kinetics were analyzed for both the EP and DP samples, with experimental data and model-predicted curves plotted over time (Figure 1a). The DP exhibited a slower digestion pattern, characterized by a higher maximum digested starch concentration (C_∞_ = 83.76) and lower rate constant (k = 0.0024). In contrast, EP demonstrated more rapid initial digestion kinetics, with a lower maximum digested starch concentration (C_∞_ = 21.50) and higher rate constant (k = 0.014). The digestion profile of EP samples showed characteristic rapid starch digestion in the early phases, followed by a plateauing effect as the reaction approached 120 min.

### 3.3. Protein Bioaccessibility

Analysis of protein hydrolysis (DH%) revealed no statistically significant differences between sample groups (Figure 1b). Both the EP and DP samples demonstrated median DH values of approximately 20%. While the DP sample exhibited a broader distribution of DH values, the overall protein bioaccessibility remained statistically comparable between the two pasta types. These findings indicate that both pasta formulations underwent similar degrees of protein hydrolysis during simulated digestion conditions.

### 3.4. Main Lipid Classes

Table 2 shows how the type of pasta, cooking, and digestion influenced pasta’s lipid profile, often resulting in reduced levels of bioactive compounds and an altered lipid composition that may impact the pasta’s nutritional properties.

In raw samples and after cooking, the most abundant lipid class was TAG, followed by DAG, FFA, MAG, STE, TOC, and E-STE. In contrast, after in vitro digestion, the most abundant class was FFA, followed by DAG, MAG, TAG, and STE; TOC and E-STE were not detected. Sample type, cooking, and digestion appear to be significant for all parameters except for TOC, where sample type did not affect its content. As for interactions, however, type × cooking was significant for all parameters analyzed, except for MAG and DAG. Finally, the type × digestion interaction was not significant for FFA and DAG.

### 3.5. Total Fatty Acids

As reported in Table 3, polyunsaturated FA (PUFA) was the most represented FA class (ranging from 58.12 to 59.67% of total FA), followed by monounsaturated FA (MUFA, ranging from 18.09 to 22.52% of total FA), and saturated FA (SFA, ranging from 17.40 to 19.13% of total FA) in raw samples. SFA content was significantly influenced by pasta type and digestion. While cooking did not significantly affect SFA content, digestion caused minor but statistically notable changes. MUFA were significantly influenced by all factors; in particular, digestion led to a sharp increase in MUFA content. PUFA were also significantly affected by all factors; in this case, both cooking and digestion had a relevant impact (*p* < 0.0001) on PUFA, with digestion causing a marked decrease. *n*-6 and essential fatty acids (EFA) were highly present in both raw and cooked pasta samples (55–63% of total FA). After cooking, *n*-6 PUFA content either slightly increased or stabilized; however, there was a substantial reduction after digestion. In particular, *n*-6 PUFA in both digested pasta samples decreased to about 35% of total FA. Regarding *n*-3 PUFA, they were relatively higher in raw and cooked pasta samples, showing 3.07% and 3.83% *n*-3 PUFA (of total FA) in raw EP and DP, respectively; moreover, cooking did not significantly affect *n*-3 PUFA content of EP and DP (3.29% and 3.98% of total FA, respectively). However, there was a sharp reduction in *n*-3 PUFA after in vitro digestion (1.64% and 2.00% of *n*-3 PUFA in EP and DP, respectively). The *n*-3 PUFA were considerably affected by all factors (the type of the sample, the cooking, and the in vitro digestion).

The PUFA/SFA ratio remained relatively stable in raw and cooked samples, with values of 3.34 and 3.12 in raw EP and DP, respectively, and it was slightly higher in cooked samples (3.41 and 3.32 for EP and DP, respectively). However, a substantial post-digestion decrease was observed in this ratio, with digested EP and DP samples dropping to 2.19 and 2.14, respectively. The UFA/SFA ratio was relatively high in pasta samples (4.07–4.81), indicating a predominance of unsaturated over saturated fats. This ratio shows statistical significance for pasta type but remains non-significant across cooking and digestion. Regarding the *n*-6/*n*-3 ratio, it was relatively low in raw and cooked pasta, ranging from 14.63 to 18.01. However, after digestion, the ratio increased, particularly in EP, where it reached 21.19. The *n*-6/*n*-3 ratio was significantly affected by the pasta type and digestion stages.

Regarding single FA, the most abundant FA in all analyzed samples was linoleic acid (C18:2), followed by oleic acid (C18:1), palmitic acid (C16:0), and α-linolenic acid (C18:3 α) (Table S2). C16:0 and C18:0 showed slight changes across cooking and digestion stages. C18:1 slightly decreased during cooking, but it significantly increased after digestion. C18:2 and C18:3 α were impacted by cooking and digestion, the latter showing a greater impact on these FA.

### 3.6. Total Sterols

The total sterols content of samples ranged between 1017 and 10,934 mg/100 g of pasta. The most abundant sterol was β-sitosterol, followed by campesterol, Δ^5^-avenasterol, sitostanol, Δ^5,24^-stigmastadienol, Δ^7^-stigmastanol, stigmasterol, and Δ^7^-avenasterol (Table 4).

β-sitosterol levels were higher in EP samples (930–3284 mg/100 g of pasta) than in DP samples (624–2452 mg/100 g of pasta). Grain type, cooking, and digestion significantly influenced β-sitosterol content, while only the type × digestion interaction turned out to be significant. Δ^5^-avenasterol, Δ^5,24^-stigmastadienol, Δ^7^-stigmastanol, stigmasterol, and Δ^7^-avenasterol showed the same trend, being consistently higher in EP samples than in DP. Their content and distribution, in fact, was influenced by all the factors tested (pasta type, cooking, and digestion), as well as their interactions. Stigmasterol content was also influenced by all the factors tested and their interactions; however, it must be noted that it was always higher in DP samples compared to EP ones.

Following in vitro digestion, total sterol content decreased substantially in both pasta types; EP retained 17% of its initial content, while DP showed 14% sterol retention.

### 3.7. Tocopherols

Table 5 shows that only α- and β-tocopherols were detected in both pasta samples before and after cooking, as well as after their in vitro digestion, with α-tocopherol being the most abundant compound. Moreover, the tocopherol content in the EP samples was 2-times higher than in DP, and this difference was kept even after being cooked and digested, despite the decrease observed in both types of pasta. In fact, after in vitro digestion, the total tocopherols content remained higher in EP samples (5.96 mg/kg of pasta), compared to DP samples (1.98 mg/kg of pasta). Type, cooking, digestion, and their interaction significantly influenced the total tocopherols content.

Both detected tocopherols were consistently higher in EP samples than in DP, but while all the factors tested and their interactions significantly influenced the α-tocopherol level, the content of β-tocopherol was not significantly influenced by the T × C interaction.

The in vitro digestion process led to different retention rates of total tocopherols: EP retained 57% of its initial content (decreasing from 9.99 to 5.69 mg/kg), while DP showed lower retention at 33% (decreasing from 6.04 to 1.98 mg/kg).

### 3.8. Phenolic Acids

The results demonstrated that total phenols in EP and DP were significantly affected by all factors (cereal type, cooking, and digestion). The raw pasta samples had a total phenol content of 38.22 mg/kg of pasta for EP samples and 18.64 mg/kg of pasta for DP samples. After cooking, the total phenols content decreased to 28.22 mg/kg of paste and 10.90 mg/kg of pasta for EP and DP samples, respectively. Finally, after in vitro digestion, the total phenol content always remained higher in EP samples (17.07 mg/kg of pasta), compared to DP samples (4.71 mg/kg of pasta).

Insoluble phenols were more abundant than soluble ones in both types of pasta samples before and after cooking, as well as after their in vitro digestion (Table 6). Among soluble phenols, syringic acid was the most abundant, followed by ferulic, coumaric, gallic, chlorogenic, and caffeic acids. The same trends were maintained also for insoluble phenols. The statistical analysis highlights that the changes in phenolic content across treatments (einkorn and durum wheat), cooking stages, and digestion phases were significant. All individual phenols (such as ferulic, coumaric, syringic, caffeic, chlorogenic, and gallic acids) showed significant reductions (*p* < 0.001) across both pasta types from raw to digested, with pronounced declines especially in the digested DP samples. In addition, the total phenol content underwent a significant decrease (*p* < 0.001), which was influenced by cereal type, cooking, and digestion factors, as well as for their interactions. Some interactions, such as cooking × digestion (C × D), were not significant for specific phenols, evidencing a compound-dependent variability in response to cooking and digestion.

The simulated digestion resulted in differential retention of phenolic compounds between pasta types: EP maintained 45% of its initial total phenolic content (decreasing from 38.22 to 17.07 mg/kg), while DP showed 25% retention (decreasing from 18.64 to 4.71 mg/kg).

### 3.9. Total Antioxidant Capacity

TAC was evaluated using two complementary assays: DPPH and FRAP. Two-way ANOVA revealed significant effects of both cereal type and cooking on antioxidant capacity (*p* < 0.0001).

**Figure 2:**
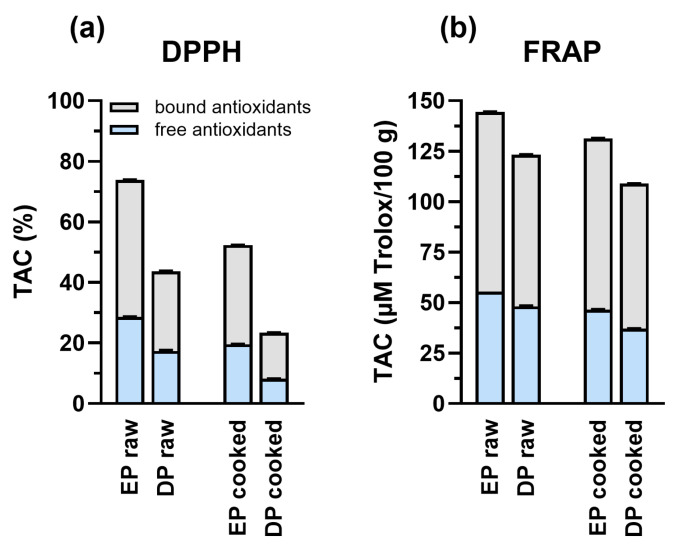
Total antioxidant capacity (TAC) of EP and DP before and after cooking, measured by two different methods. (**a**) DPPH (2,2′-diphenyl-1-picrylhydrazyl) radical scavenging activity expressed as percentage of total antioxidant capacity (TAC%). (**b**) FRAP (Ferric Reducing Antioxidant Power) assay results expressed as μM Trolox/100 g. Both panels show the distribution between free antioxidants (light blue bars) and bound antioxidants (grey bars). Two-way ANOVA showed significant effects of cereal type (DPPH: F(3,19) = 147.0, *p* < 0.0001; FRAP: F(3,19) = 61.03, *p* < 0.0001) and cooking (DPPH: F(1,19) = 175.8, *p* < 0.0001; FRAP: F(1,19) = 1229, *p* < 0.0001) on TAC.

For the DPPH assay (Figure 2a), EP samples showed higher radical scavenging activity compared to DP in both raw and cooked samples. Free antioxidants, that are readily extractable, accounted for about 30–40% of the total TAC, while bound antioxidants, that are associated with the food matrix, accounted for 60–70%. Cooking resulted in a general decrease in TAC for both pasta types, with EP retaining higher activity after cooking compared to DP.

FRAP assay results showed similar trends (Figure 2b), with EP exhibiting higher iron reducing power than DP. Raw EP showed approximately 140 μM Trolox/100 g TAC, with an approximately equal distribution between free and bound antioxidants. Cooking caused a moderate decrease in FRAP values for both types of pasta, even though EP maintained a higher antioxidant capacity throughout processing.

### 3.10. Prebiotic Evaluation

The addition of homogenized pasta samples (10%, *w*/*v*) in saline solution to culture medium showed differential effects on microbial growth. Lactobacilli mix growth remained unchanged compared to the glucose negative control for both EP and DP samples. However, the bifidobacteria mix exhibited enhanced growth, increasing by 3.4 Log units with pasta additions. Notably, EP demonstrated significantly higher growth promotion compared to DP, matching the positive control (FOS) effectiveness. This differential response suggests superior prebiotic potential of EP for bifidobacteria populations. For the *Escherichia coli* mix, both the EP and DP samples supported growth to 3.7 Log units, showing no significant difference from controls (glucose or FOS) or between pasta types (Figure S1).

Prebiotic scoring revealed distinct patterns across bacterial groups. FOS demonstrated prebiotic scores of 0.37 and 0.33 towards lactobacilli and bifidobacteria, respectively (Table 7). EP showed moderate prebiotic activity towards lactobacilli (0.18), while DP showed inhibitory effects (−0.30). Similarly, EP exhibited prebiotic activity towards bifidobacteria (0.26), whereas DP showed slight inhibition (−0.09).

**Figure 3:**
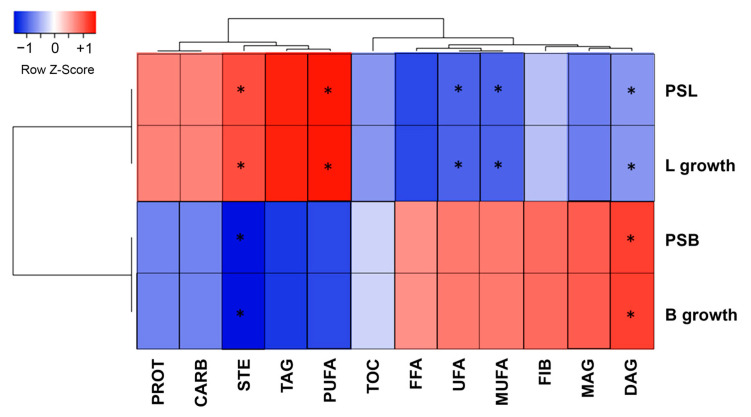
Correlations of prebiotic activity and class of molecules from the nutritional composition of pasta products. PROT = protein content; CARB = carbohydrate content; STE = sterols content; TAG = triacylglycerol content; PUFA = polyunsaturated fatty acids content; TOC = tocopherol content; FFA = free fatty acid content; UFA = unsaturated fatty acid; MUFA = monounsaturated fatty acid content; FIB = fiber content; MAG = monoacylglycerol content; DAG = diacylglycerol content; PSL = prebiotic activity of lactobacilli mix; L growth = selective growth of lactobacilli mix; PSB = prebiotic activity of bifidobacteria mix; B growth = selective growth of bifidobacteria mix. Correlation marked with * has statistical significance at *p* < 0.05.

Correlation analysis revealed distinct nutritional associations with bacterial growth. Lactobacilli prebiotic activity positively correlated with protein, carbohydrate, and TAG content, with significant correlations to PUFA and STE, supporting previous findings linking lactobacilli growth to PUFA [84]. Bifidobacteria growth and prebiotic scores showed positive correlations with FFA, UFA, MUFA, fiber, and MAG, with particularly significant correlation to DAG content (Figure 3). This aligns with previous research showing DAG’s role in promoting bifidobacterial growth [85].

## 4. Discussion

The comparative analysis of wholegrain einkorn pasta (EP) and durum wheat pasta (DP) revealed distinct compositional and functional characteristics that persisted through cooking and in vitro digestion. While EP is produced by stone-grinding the wholegrain kernel, preserving the natural integration of bran and germ components, DP is manufactured through industrial milling where bran is first separated and then reincorporated [32,33].

Proximate analysis demonstrated similar macronutrient profiles between the two pasta types, with comparable energy content and nutrient levels (Table 1). Notably, both products contained over 6 g fiber/100 g, qualifying them for “high in fiber” claims under European Regulation No. 1924/2006 [86]. This significant fiber content has important health implications, as dietary fiber intake has been linked to reduced risk of chronic diseases like colorectal cancer [87,88] and improved gut microbiota diversity [89,90]. Although ancient wheat varieties typically exhibit higher mineral content compared to modern cultivars [5,71], neither pasta type achieved the thresholds for “source” or “high” mineral content claims under European Regulation No. 1924/2006 [15% RDA for iron (14 mg) or 30% RDA for zinc (15 mg)] [86]. This finding suggests that milling and pasta production techniques may influence final mineral content more significantly than wheat variety selection [23].

Protein bioaccessibility remained comparable between varieties, with both showing hydrolysis degrees around 20% (Figure 1b), within expected ranges for pasta products [91]. However, this value likely represents a much lower protein bioaccessibility than would occur in vivo, as the INFOGEST protocol lacks BBM enzymes. BBM contains more than twenty different enzymes, with peptidases (including exopeptidases, endopeptidases, enteropeptidases, and dipeptidases) and di(oligo)saccharidases being the most abundant [92], which would significantly enhance protein breakdown during intestinal digestion. Specifically, BBM peptidases are not yet commercially available, limiting their inclusion in in vitro digestion protocols [93].

While protein bioaccessibility was similar between EP and DP, the products showed distinct patterns in starch bioaccessibility (Figure 1a). EP demonstrated more rapid initial starch hydrolysis with a higher rate constant (k), while DP exhibited slower digestion kinetics without reaching a plateau during AMG hydrolysis. These differences in starch bioaccessibility reflected distinct susceptibilities to enzymatic breakdown during gastrointestinal digestion [94], likely due to the different nature of bran incorporation in the two products. Einkorn protein composition leads to softer dough with lower elasticity compared to common wheat [95], contributing to unique functional properties like enhanced starch accessibility. The einkorn flour doughs have higher gliadin-to-glutenin ratios, which impact both processing requirements and final product characteristics [14]. This fundamental difference in processing bran components results in diverse structural organizations of the food matrix, significantly impacting glucose release during digestion. In addition, our results align with those reported by Dodi et al. (2021) [96], who observed similar percentages of starch digestion at 120 min in pasta samples with different fiber content.

The different bran incorporation methods influenced prebiotic potential through fiber components. Specifically, arabinoxylans and other fiber constituents modulate gut microbiota composition [97]. EP demonstrated enhanced prebiotic activity, especially toward bifidobacteria populations, matching the effectiveness of fructooligosaccharide controls (Table 7). This superior prebiotic effect stemmed from EP’s preserved grain structure, as ancient wheat varieties contain varying concentrations of prebiotic compounds including fructans, arabinoxylans, resistant starch, and β-glucans [20]. Further supporting these findings, correlation analysis revealed specific associations between pasta composition and bacterial growth (Figure 3). Bifidobacteria growth showed strong positive correlations with fiber content and diacylglycerol levels [85], while lactobacilli activity correlated with protein, carbohydrate, and polyunsaturated fatty acid content [84]. These relationships suggest that EP’s intact grain structure and preserved bioactive compounds contribute to its enhanced prebiotic functionality.

The diverse bran incorporation methods also significantly influenced lipid profiles in both pasta types (Table 2). STE concentrations remained relatively unchanged in DP while their content was reduced in EP. Its reduction could be attributed to sterol oxidation or dehydration [98]. During cooking, lipolysis predictably reduced TAG content while increasing partial glycerides and FFA. Both products showed decreased SFA and MUFA during cooking (Table 3), likely due to physical entrapment within the protein–starch network formed during gelatinization, where amylose–lipid complexes and protein–lipid interactions create a dense matrix limiting lipid accessibility [99,100]. Conversely, during gastrointestinal digestion, PUFA liberation from the food matrices was significantly reduced (Table 3), attributed to their preferential esterification at the *sn*-2 position of the TAG molecule, while pancreatic lipase exhibits regiospecificity for primary ester bonds at the *sn*-1 and *sn*-3 positions [101].

Despite similar fatty acid profiles, EP demonstrated superior retention of sterols, particularly β-sitosterol, throughout processing and digestion (Table 4), reflecting einkorn’s distinctive characteristics and higher germ-to-endosperm ratio [102]. The significant release of sterols during enzymatic hydrolysis has also been verified in other cereals subjected to in vitro digestion [67] and suggests potential intestinal absorption, which could contribute to a cholesterol lowering effect [103].

Additionally, EP exhibited higher levels of α- and β-tocopherols compared to DP (Table 5). Although cooking decreased tocopherol content in both samples through heat-induced degradation, EP maintained consistently higher concentrations [7], potentially offering enhanced antioxidant protection. Analysis of retention rates after digestion showed differential stability of bioactive compounds between the two pasta types: EP showed markedly higher retention for bioactive compounds compared to DP (tocopherols 57% vs. 33%, phenolics 45% vs. 25%, and sterols 17% vs. 14%) (Table 4, Table 5 and Table 6). This superior retention of bioactive compounds suggests that EP’s matrix may help to better preserve these beneficial components during both thermal processing and digestion.

The enhanced preservation of bioactive compounds was further evidenced by EP’s superior phenolic content (Table 6) and antioxidant capacity throughout processing and digestion; ancient grains like spelt tend to retain higher levels of bioactive compounds, such as phenolics, compared to more intensively farmed modern varieties [3,13,104]. The phenolic profile included antioxidants such as ferulic, syringic, and coumaric acids, which play key roles in preventing oxidative stress [105,106]. EP’s higher proportion of soluble phenols (Table 6) is particularly significant for nutritional value, as these compounds show greater accessibility compared to insoluble forms bound within the fiber matrix [107,108].

Finally, bound antioxidants accounted for the majority of total antioxidant activity in both pasta types (Figure 2), characteristic of wholegrain cereals [109,110]. This arrangement enables gradual release through enzymatic digestion [30]. Both DPPH (Figure 2a) and FRAP assays (Figure 2b) confirmed that EP’s superior antioxidant capacity persisted after cooking, suggesting enhanced stability of these compounds in the einkorn matrix.

While this study provides valuable insights into einkorn pasta’s properties through cooking and digestion, some limitations should be acknowledged. Our analysis focused specifically on einkorn pasta, though the methodological framework could be readily applied to other ancient grains like emmer and spelt for comparative evaluation with conventional durum products. While one pasta format (penne) was examined, this analytical approach could be extended to various pasta shapes and other cereal-derived products, made either from flour or semolina, wholegrain or refined. In addition, microscopic analysis would be needed to fully elucidate the exact mechanisms underlying the observed differences in bioactive compound retention and matrix–digestive enzyme interactions (e.g., microstructural organization of starch, protein, and fiber components).

## 5. Conclusions

These findings demonstrate einkorn pasta’s potential as a functional food ingredient, with its unique stone-ground processing method preserving the natural integration of bran and germ components, leading to enhanced retention of bioactive compounds. While einkorn pasta showed more rapid initial starch digestion kinetics compared to conventional durum wheat pasta, the higher levels of phenolics, tocopherols, and sterols, combined with improved prebiotic activity and antioxidant capacity, suggest potential health benefits beyond basic nutrition.

The results have important implications for pasta manufacturing, indicating that traditional processing methods can be leveraged to create nutritionally enhanced products while maintaining conventional pasta characteristics.

Future research should focus on innovative processing technologies, including optimized extrusion parameters, to enhance beneficial compound retention while maintaining pasta quality. Development of formulation strategies combining einkorn with complementary ingredients such as other ancient cereal or legume flours could further enhance both nutritional and technological properties. In addition, human intervention studies are needed to validate the observed prebiotic effects and examine how einkorn pasta consumption influences gut microbiota composition, metabolic health markers, and bioactive compound bioavailability. These research directions will collectively contribute to establishing einkorn pasta as a commercially viable functional food product while promoting crop diversity and sustainable food system development.

## Figures and Tables

**Table 1:** Nutrient composition of pasta, per 100 g. Data are reported as mean ± SD (standard deviation). *p* values were reported from comparisons by unpaired *t*-test.

Nutrient/Energy	EP	DP	*p* Value
Energy (kJ)	1467 ± 20	1492 ± 3	0.1009
Protein (g)	14.02 ± 0.76	14.53 ± 0.48	0.3827
Carbohydrates (g)	64.13 ± 0.42	64.48 ± 0.62	0.4679
Sugars, total (g)	2.54 ± 0.09	2.45 ± 0.20	0.5193
Fiber, total (g)	8.01 ± 0.53	7.47 ± 0.19	0.1728
Fat (g)	2.00 ± 0.14	2.40 ± 0.38	0.1613
Ash (g)	1.76 ± 0.16	1.62 ± 0.11	0.2673
Water (g)	10.08 ± 0.60	9.51 ± 0.37	0.2308
Salt (g)	0.01 ± 0.00	0.01 ± 0.00	0.6937
Zinc (mg)	5.03 ± 0.45	5.29 ± 0.89	0.6793
Iron (mg)	4.38 ± 0.35	4.54 ± 0.55	0.6868

**Table 2:** Main lipid classes profile of raw, cooked, and digested pasta samples.

Lipid Classes	Raw		Cooked		Digested		p Value				
	EP	DP	EP	DP	EP	DP	T	C	D	T × C	T × D
(% of Total Lipids)											
**FFA**	12.66 ± 0.17 ^A,c^	3.15 ± 0.08 ^B,e^	12.05 ± 0.63 ^A,c^	5.41 ± 0.30 ^B,d^	65.29 ± 0.91 ^A,a^	59.65 ± 0.65 ^B,b^	***	*	***	**	*ns*
**MAG**	3.63 ± 0.15 ^A,e^	1.90 ± 0.15 ^B,f^	7.40 ± 0.63 ^A,c^	5.20 ± 0.24 ^B,d^	10.52 ± 0.71 ^B,b^	19.43 ± 0.48 ^A,a^	***	***	***	*ns*	***
**TOC**	0.43 ± 0.02 ^A,c^	0.44 ± 0.02 ^A,c^	0.87 ± 0.04 ^A,a^	0.76 ± 0.05 ^B,b^	0.00 ± 0.00 ^A,a^	0.00 ± 0.00 ^A,d^	*ns*	***	***	**	**
**STE**	3.16 ± 0.03 ^A,a^	2.61 ± 0.10 ^B,b^	1.97 ± 0.03 ^B,c^	2.67 ± 0.14 ^A,b^	0.09 ± 0.00 ^A,d^	0.06 ± 0.00 ^B,d^	***	***	***	***	***
**E-STE**	0.04 ± 0.00 ^A,b^	0.00 ± 0.00 ^B,c^	0.00 ± 0.00 ^B,c^	0.09 ± 0.01 ^A,a^	0.00 ± 0.00 ^A,c^	0.00 ± 0.00 ^A,c^	***	***	***	***	***
**DAG**	19.12 ± 0.28 ^A,c^	13.13 ± 0.50 ^B,e^	21.23 ± 1.55 ^A,b^	16.84 ± 0.45 ^B,d^	23.26 ± 0.10 ^A,a^	20.12 ± 0.08 ^B,bc^	***	***	***	*ns*	*ns*
**TAG**	60.87 ± 0.25 ^B,c^	78.75 ± 0.77 ^A,a^	56.52 ± 1.98 ^B,d^	69.04 ± 0.83 ^A,b^	0.63 ± 0.02 ^B,e^	0.80 ± 0.02 ^A,e^	***	***	***	**	***

Results as reported as mean ± SD of 3 independent replicates. ^A,B^ indicates significant differences (Tukey’s test; *p <* 0.05) between DP, wholegrain durum wheat pasta, and EP, einkorn pasta samples; ^a–f^ indicates significant differences (Tukey’s test; *p* < 0.05) between samples within the same lipid class. * *p* < 0.05, ** *p* < 0.01, *** *p* < 0.0001. *ns*, not significant. C, cooking; D, digestion; FFA, free fatty acids; MAG, monoacylglycerols; T, type; TOC, tocopherols; STE, sterols; E-STE, esterified sterols; DAG, diacylglycerols; TAG, triacylglycerols.

**Table 3:** Main FA classes profile of raw, cooked, and digested pasta samples.

FA Classes	Raw		Cooked		Digested		*p* Value				
	EP	DP	EP	DP	EP	DP	T	C	D	T × C	T × D
(% of Total Fatty Acids)											
**SFA**	17.40 ± 0.16 ^B,bc^	19.13 ± 0.43 ^A,a^	17.96 ± 0.42 ^B,abc^	19.04 ± 0.32 ^A,ab^	16.60 ± 0.50 ^B,c^	17.87 ± 1.25 ^A,abc^	**	*ns*	**	*ns*	*ns*
**MUFA**	22.52 ± 0.42 ^A,c^	18.09 ± 0.24 ^B,d^	19.20 ± 0.07 ^A,d^	16.56 ± 0.17 ^B,d^	45.40 ± 1.89 ^A,a^	38.01 ± 1.72 ^B,b^	***	**	***	*ns*	*
**PUFA**	58.12 ± 0.32 ^A,b^	59.67 ± 0.54 ^A,ab^	61.24 ± 0.31 ^B,ab^	63.24 ± 0.23 ^A,a^	38.36 ± 1.46 ^B,c^	38.05 ± 2.79 ^A,c^	*ns*	**	***	*ns*	*ns*
***n*-6**	55.23 ± 0.27 ^A,b^	55.95 ± 0.58 ^A,ab^	58.09 ± 0.27 ^B,ab^	59.37 ± 0.19 ^A,a^	34.72 ± 1.44 ^B,c^	36.05 ± 2.73 ^A,c^	*ns*	**	***	*ns*	*ns*
***n*-3**	3.07 ± 0.11 ^B,c^	3.83 ± 0.06 ^A,a^	3.29 ± 0.03 ^B,b^	3.98 ± 0.04 ^A,a^	1.64 ± 0.02 ^B,e^	2.00 ± 0.07 ^A,d^	***	**	***	*ns*	**
**EFA**	58.12 ± 0.32 ^B,b^	59.67 ± 0.54 ^A,ab^	61.24 ± 0.31 ^B,ab^	63.24 ± 0.23 ^A,a^	38.36 ± 1.46 ^B,c^	38.05 ± 2.79 ^A,c^	*ns*	**	***	*ns*	*ns*
**PUFA/SFA**	3.34 ± 0.03 ^A,a^	3.12 ± 0.09 ^B,a^	3.41 ± 0.10 ^A,a^	3.32 ± 0.06 ^B,a^	2.31 ± 0.02 ^A,b^	2.14 ± 0.23 ^A,b^	*ns*	*ns*	***	*ns*	*ns*
**UFA/SFA**	4.63 ± 0.05 ^A,ab^	4.07 ± 0.12 ^B,c^	4.48 ± 0.13 ^A,abc^	4.19 ± 0.09 ^B,bc^	4.81 ± 0.17 ^A,a^	4.28 ± 0.42 ^A,abc^	***	*ns*	*ns*	*ns*	*ns*
***n*-6/** ***n*-3**	18.01 ± 0.65 ^A,b^	14.63 ± 0.35 ^B,c^	17.64 ± 0.06 ^A,b^	14.92 ± 0.11 ^B,c^	21.19 ± 0.60 ^A,a^	18.01 ± 0.76 ^B,b^	***	*ns*	***	*ns*	*ns*

Results as reported as mean ± SD of 3 independent replicates. ^A,B^ indicates significant differences (Tukey’s test; *p* ≤ 0.05) between DP, whole meal durum wheat pasta, and EP, einkorn pasta samples. ^a–e^ indicates significant differences (Tukey’s test; *p* ≤ 0.05) between samples within the same FA class. * *p* < 0.05, ** *p* < 0.01, *** *p* < 0.0001. *ns*, not significant. C, cooking; D, digestion; EFA, essential fatty acids; MUFA, monounsaturated fatty acids; PUFA, polyunsaturated fatty acids; SFA, saturated fatty acids; T, type.

**Table 4:** Main sterols profile of raw, cooked, and digested pasta samples.

Sterols	Raw		Cooked		Digested		*p* Value				
	EP	DP	EP	DP	EP	DP	T	C	D	T × C	T × D
	(mg/100 g of Pasta)										
**Campesterol**	1166.87 ± 13.93 ^A,a^	851.73 ± 28.40 ^B,c^	948.96 ± 17.88 ^A,b^	562.60 ± 9.81 ^C,d^	330.40 ± 8.25 ^A,e^	128.56 ± 5.01 ^B,f^	***	***	***	**	***
**Campestanol**	13.85 ± 0.03 ^A,a^	13.82 ± 0.01 ^A,a^	13.87 ± 0.02 ^A,a^	13.87 ± 0.05 ^A,a^	2.70 ± 0.16 ^A,b^	1.70 ± 0.34 ^B,c^	***	*ns*	***	*ns*	***
**Stigmasterol**	225.52 ± 2.87 ^B,b^	331.71 ± 12.55 ^A,a^	156.62 ± 1.21 ^B,d^	195.60 ± 2.10 ^A,c^	37.21 ± 2.29 ^B,e^	40.09 ± 0.09 ^A,e^	***	***	***	***	***
**β-sitosterol**	3284.88 ± 17.22 ^A,a^	2452.10 ± 149.06 ^B,b^	2254.22 ± 52.18 ^A,b^	1393.17 ± 92.04 ^B,c^	930.00 ± 11.98 ^A,d^	624.35 ± 6.34 ^B,e^	***	***	***	*ns*	***
**Sitostanol**	632.25 ± 10.27 ^A,a^	397.96 ± 23.72 ^B,b^	385.80 ± 17.95 ^A,b^	208.97 ± 50.65 ^B,c^	218.09 ± 13.78 ^A,c^	102.70 ± 4.20 ^B,d^	***	***	***	*ns*	*ns*
**Δ^5^-avenasterol**	1068.11 ± 21.08 ^A,a^	387.86 ± 13.14 ^B,c^	702.70 ± 42.87 ^A,b^	224.30 ± 4.52 ^B,d^	173.59 ± 16.61 ^A,d^	68.32 ± 1.56 ^B,e^	***	***	***	***	***
**Δ^5,24^-stigmastadienol**	445.36 ± 1.07 ^A,a^	48.64 ± 4.85 ^B,d^	259.06 ± 5.42 ^A,b^	16.30 ± 1.41 ^B,e^	75.50 ± 3.08 ^A,c^	8.48 ± 0.45 ^B,e^	***	***	***	***	***
**Δ^7^-stigmastanol**	495.01 ± 33.69 ^A,a^	297.92 ± 28.92 ^B,b^	460.85 ± 3.62 ^A,a^	161.14 ±10.68 ^B,c^	78.41 ± 1.69 ^A,d^	32.06 ± 1.40 ^B,d^	***	***	***	**	***
**Δ^7^-avenasterol**	147.61 ± 2.52 ^A,a^	85.76 ± 0.49 ^B,c^	125.89 ± 2.06 ^A,b^	23.27 ± 0.30 ^B,e^	32.44 ± 1.22 ^A,d^	5.60 ± 0.46 ^B,f^	***	***	***	***	***
**Total sterols**	10,934.73 ± 8.14 ^A,a^	7183.99 ± 141.54 ^B,c^	2526.11 ± 136.59 ^A,b^	1545.59 ± 132.30 ^B,d^	1878.34 ± 47.46 ^A,e^	1017.63 ± 19.40 ^B,f^	***	***	***	*ns*	***

Results as reported as mean ± SD of 3 independent replicates. ^A,B,C^ indicates significant differences (Tukey’s test; *p <* 0.05) between DP, wholegrain durum wheat pasta, and EP, einkorn pasta samples. ^a–f^ indicates significant differences (Tukey’s test; *p* < 0.05) between samples. *ns*, not significant. DP, wholegrain durum wheat pasta; EP, einkorn pasta. ** *p* < 0.01, *** *p* < 0.0001. *ns*, not significant. C, cooking; D, digestion; T, type.

**Table 5:** Main tocopherols of raw, cooked, and digested pasta samples.

Tocopherols	Raw		Cooked		Digested		*p* Value				
	EP	DP	EP	DP	EP	DP	T	C	D	T × C	T × D
(mg/kg of Pasta)											
**α-tocopherol**	7.88 ± 0.09 ^A,a^	4.67 ± 0.14 ^B,c^	7.02 ± 0.12 ^A,b^	2.22 ± 0.09 ^B,d^	4.70 ± 0.34 ^A,c^	1.41 ± 0.13 ^B,e^	***	***	***	***	***
**β-tocopherol**	2.12 ± 0.06 ^A,a^	1.37 ± 0.07 ^B,c^	1.90 ± 0.01 ^A,b^	1.07 ± 0.04 ^B,d^	0.99 ± 0.03 ^A,d^	0.57 ± 0.03 ^B,e^	***	***	***	*ns*	***
**Total**	9.99 ± 0.02 ^A,a^	6.04 ± 0.05 ^B,c^	8.92 ± 0.08 ^A,b^	3.30 ± 0.03 ^B,d^	5.69 ± 0.14 ^A,c^	1.98 ± 0.10 ^A,e^	***	***	***	*	***

Results as reported as mean ± SD of 3 independent replicates. ^A,B^ indicates significant differences (Tukey’s test; *p* < 0.05) between DP, wholegrain durum wheat pasta, and EP, einkorn pasta samples. ^a–e^ indicates significant differences (Tukey’s test; *p* < 0.05) between samples. * *p* < 0.05, *** *p* < 0.0001. *ns*, not significant. C, cooking; D, digestion; T, type.

**Table 6:** Main phenols profile (soluble and insoluble) of raw, cooked, and digested pasta samples.

Phenols		Raw		Cooked		Digested		*p* Value				
		EP	DP	EP	DP	EP	DP	T	C	D	T × C	T × D
	(mg/kg of pasta)											
**Soluble phenols**	**Ferulic**	4.57 ± 0.24 ^A,a^	1.92 ± 0.06 ^B,c^	3.81 ± 0.14 ^A,b^	0.75 ± 0.05 ^B,d^	1.72 ± 0.16 ^A,c^	0.35 ± 0.03^B,e^	***	***	***	*	***
	**Coumaric**	4.08 ± 0.05 ^A,a^	2.25 ± 0.12 ^B,c^	3.53 ± 0.34 ^A,b^	0.66 ± 0.04 ^B,e^	1.59 ± 0.15 ^A,d^	0.25 ± 0.02^B,e^	***	***	***	***	***
	**Syringic**	4.73 ± 0.11 ^A,a^	2.31 ± 0.15 ^B,d^	3.72 ± 0.14 ^A,b^	1.11 ± 0.02 ^B,e^	2.67 ± 0.17 ^A,c^	0.58 ± 0.03^B,f^	***	***	***	*ns*	**
	**Caffeic**	0.69 ± 0.02 ^A,a^	0.25 ± 0.03 ^B,c^	0.42 ± 0.02 ^A,b^	0.14 ± 0.01 ^B,d^	0.13 ± 0.01 ^A,d^	0.00 ± 0.00^B,e^	***	***	***	***	***
	**Chlorogenic**	0.71 ± 0.01 ^A,a^	0.21 ± 0.01 ^B,c^	0.26 ± 0.03 ^A,b^	0.07 ± 0.01 ^B,e^	0.13 ± 0.01 ^A,d^	0.00 ± 0.00^B,f^	***	***	***	***	**
	**Gallic**	1.46 ± 0.11 ^A,a^	1.27 ± 0.11 ^B,b^	0.51 ± 0.02 ^A,c^	0.45 ± 0.04 ^B,cd^	0.27 ± 0.02 ^A,de^	0.15 ± 0.02^B,e^	**	***	***	*ns*	*ns*
	**Total**	16.24 ± 0.42 ^A,a^	8.21 ± 0.28 ^B,c^	12.24 ± 0.49 ^A,b^	3.18 ± 0.08 ^B,e^	6.51 ± 0.41 ^A,d^	1.33 ± 0.03^B,f^	***	***	***	**	***
**Insoluble phenols**	**Ferulic**	5.90 ± 0.05 ^A,a^	2.15 ± 0.02 ^B,d^	4.35 ± 0.19 ^A,b^	1.95 ± 0.07 ^B,d^	2.44 ± 0.12 ^A,c^	1.00 ± 0.03^B,e^	***	***	***	***	***
	**Coumaric**	5.38 ± 0.15 ^A,a^	2.60 ± 0.16 ^B,d^	4.81 ± 0.18 ^A,b^	1.78 ± 0.13 ^B,e^	3.11 ± 0.04 ^A,c^	0.81 ± 0.02^B,f^	***	***	***	*ns*	**
	**Syringic**	6.11 ± 0.13 ^A,a^	2.48 ± 0.16 ^B,d^	4.25 ± 0.08 ^A,b^	2.27 ± 0.16 ^B,d^	3.26 ± 0.08 ^A,c^	1.09 ± 0.07^B,e^	***	***	***	***	*ns*
	**Caffeic**	1.35 ± 0.08 ^A,a^	0.91 ± 0.03 ^B,b^	0.94 ± 0.05 ^A,b^	0.56 ± 0.05 ^B,c^	0.32 ± 0.01 ^A,d^	0.12 ± 0.01^B,e^	***	***	***	*ns*	**
	**Chlorogenic**	1.32 ± 0.06 ^A,a^	0.60 ± 0.03 ^B,b^	0.57 ± 0.03 ^A,b^	0.41 ± 0.03 ^B,c^	0.36 ± 0.04 ^A,c^	0.11 ± 0.01^B,d^	***	***	***	***	*ns*
	**Gallic**	1.92 ± 0.05 ^A,a^	1.70 ± 0.10 ^B,b^	1.06 ± 0.04 ^A,c^	0.75 ± 0.06 ^B,d^	1.08 ± 0.03 ^A,c^	0.25 ± 0.05^B,e^	***	***	***	*ns*	***
	**Total**	21.98 ± 0.29 ^A,a^	10.43 ± 0.06 ^B,c^	15.98 ± 0.51 ^A,b^	7.72 ± 0.24 ^B,d^	10.56 ± 0.23 ^A,c^	3.38 ± 0.14^B,e^	***	***	***	***	**
**Total phenols**		38.22 ± 0.88^A,a^	18.64 ± 0.34 ^B,c^	28.22 ± 0.67 ^A,b^	10.90 ± 0.04 ^B,d^	17.07 ± 0.35 ^A,c^	4.71 ± 0.03 ^B,e^	***	***	***	**	***

Results as reported as mean ± SD of 3 independent replicates. ^A,B^ indicates significant differences (Tukey’s test; *p <* 0.05) between DP, wholegrain durum wheat pasta, and EP, einkorn pasta samples. ^a–f^ indicates significant differences (Tukey’s test; *p* < 0.05) between samples. * *p* < 0.05, ** *p* < 0.01, *** *p* < 0.0001. *ns*, not significant. C, cooking; D, digestion; T, type.

**Table 7:** Prebiotic activity of wholegrain einkorn pasta (EP) and wholegrain durum wheat pasta (DP) compared to fructooligosaccharides (FOS) on growth of probiotic bacteria mixes.

Samples and Bacterial Populations	Prebiotic Score (mean ± SD)	*p* Value
Lactobacilli mix		
DP	−0.30 ± 0.03 ^b^	0.000455
EP	0.18 ± 0.04 ^a^	0.073431
FOS	0.37 ± 0.02 ^a^	0.002352
Bifidobacteria mix		
DP	−0.09 ± 0.02 ^b^	0.123766
EP	0.26 ± 0.04 ^a^	0.000372
FOS	0.33 ± 0.01 ^a^	0.000433

Different superscript letters within each bacterial group indicate significant differences by ANOVA followed by Tukey’s post hoc test (*p* < 0.05).

## Data Availability

The original contributions presented in the study are included in the article/Supplementary Materials, further inquiries can be directed to the corresponding author.

## Supplementary Materials

The following supporting information can be downloaded at https://www.mdpi.com/article/10.3390/foods14030370/s1: Table S1: Primers pairs used in this study [111,112,113]; Table S2: Main fatty acids profile of raw, cooked and digested pasta samples; Figure S1: Selective bacterial growth on cooked pasta.

## Abbreviations

The following abbreviations are used in this manuscript:

AACC	American Association of Cereal Chemists
AMG	Amyloglucosidase
AOAC	Association of Official Agricultural Chemists
BBM	Brush border membrane
DAG	Diacylglycerols
DH	Degree of hydrolysis
DP	Wholegrain durum wheat pasta
DPPH	2,2′-Diphenyl-1-picrylhydrazyl
E-STE	Esterified sterols
EFA	Essential fatty acids
EP	Wholegrain einkorn pasta
FA	Fatty acids
FFA	Free fatty acids
FOS	Fructooligosaccharides
FRAP	Ferric reducing antioxidant power
GC-FID	Gas chromatography–flame ionization detection
GC-MS	Gas chromatography–mass spectrometry
GOPOD	Glucose oxidase–peroxidase
HPLC-DAD	High-performance liquid chromatography coupled to a diode array detector
HPLC-FD	High-performance liquid chromatography coupled to a fluorimeter
MAG	Monoacylglycerols
MRS	Man, Rogosa, and Sharpe
MUFA	Monounsaturated fatty acids
OPA	o-phthalaldehyde
PCR	Polymerase chain reaction
PUFA	Polyunsaturated fatty acids
qPCR	Quantitative polymerase chain reaction
SDS	Sodium dodecyl sulfate
SFA	Saturated fatty acids
SIM	Single-ion monitoring
STE	Free sterols
TAC	Total antioxidant capacity
TAG	Triacylglycerols
TIC	Total ion current
TOC	Tocopherols
UFA	Unsaturated fatty acids
