# Supplementary material for: Nutritional, Chemical, and Functional Properties of Wholegrain Einkorn Pasta Through Cooking and Digestion: A Comparative Study with Wholegrain Durum Wheat Pasta
Source: Foods. 2025 Jan 23;14(3):370. doi: 10.3390/foods14030370 (PMC11817464; doi:10.3390/foods14030370)
Supplement: Supplementary file 1 [file foods-14-00370-s001.zip › foods-3412615-supplementary.pdf]

Supplementary Materials

Mercatante et al. Nutritional, Chemical and Functional Properties of Wholegrain Einkorn Pasta through Cooking and Digestion: A Comparative Study with Wholegrain Durum Wheat Pasta

Table S1. Primers pairs used in this study.

Mix target	Gene target	Sequence 5′-3′	Reference
Bifidobacteria	RecA r-RNA	fw: CGTYTCBCAGCCGGAYAAC<br>rev: CCARVGCRCCGGTCATC	Masco et al. (2007) [111]
Lactobacilli	16S V3/V4 r-RNA	fw: GCAGCAGTAGGGAATCT<br>rev: GCATTYCACCGCTACACA	Walter et al. (2000) [112]
Escherichia coli	16S V3/V4 r-RNA	fw: GTTAATACCTTTGCTCATTGA<br>rev: ACCAGGGTATCTAATCCTGTT	Malinen et al. (2003) [113]

Table S2. Main fatty acids profile of raw, cooked and digested pasta samples.

Fatty acids	Raw		Cooked		Digested		*p* Value				
	EP	DP	EP	DP	EP	DP	T	C	D	T×C	T×D
	(% of total fatty acids)										
**C16:0**	15.48±0.21^{B,b}	17.27±0.34^{A,a}	16.34±0.17^{B,ab}	17.47±0.19^{A,a}	12.15±0.32^{B,c}	13.47±1.19^{A,c}	**	*ns*	***	*ns*	*ns*
**C18:0**	1.40±0.05^{A,b}	1.51±0.13^{A,b}	1.16±0.11^{A,b}	1.22±0.10^{A,b}	3.62±0.19^{A,a}	3.78±0.16^{A,a}	*ns*	**	***	*ns*	*
**C18:1**	21.60±0.42^{A,c}	17.49±0.19^{B,d}	18.42±0.08^{A,d}	15.89±0.24^{B,d}	42.18±1.84^{A,a}	36.90±1.71^{B,b}	***	**	***	*ns*	*
**C18:2**	55.05±0.27^{A,b}	55.84±0.57^{A,ab}	57.95±0.28^{B,ab}	59.26±0.20^{A,a}	34.72±1.44^{A,c}	36.05±2.73^{A,c}	*ns*	**	***	*ns*	*ns*
**C20:0**	0.18±0.01^{A,b}	0.11±0.01^{B,cd}	0.15±0.02^{A,bc}	0.10±0.02^{B,d}	0.26±0.01^{A,a}	0.25±0.02^{A,a}	**	*	***	*ns*	*ns*
**C18:3 α linolenic**	3.07±0.11^{B,c}	3.83±0.06^{A,a}	3.29±0.03^{A,b}	3.98±0.04^{A,a}	1.64±0.02^{B,e}	2.00±0.07^{A,d}	***	**	***	*ns*	**
**C20:1**	0.84±0.03^{A,a}	0.55±0.06^{B,c}	0.70±0.02^{A,b}	0.54±0.02^{B,c}	0.58±0.00^{A,c}	0.43±0.02^{B,d}	***	**	***	**	*ns*
**C22:0**	0.20±0.01^{A,ab}	0.13±0.02^{B,bc}	0.19±0.03^{A,ab}	0.08±0.01^{B,c}	0.23±0.00^{A,a}	0.22±0.02^{A,a}	*	*ns*	***	*ns*	**
**C22:1**	0.08±0.01^{A,bc}	0.04±0.01^{B,c}	0.08±0.01^{B,bc}	0.13±0.07^{A,ab}	0.17±0.01^{A,a}	0.00±0.00^{B,c}	***	*	*ns*	*	***
**C24:0**	0.13±0.03^{A,a}	0.11±0.03^{A,a}	0.17±0.09^{A,a}	0.16±0.04^{A,a}	0.17±0.01^{A,a}	0.13±0.01^{B,a}	*ns*	*ns*	*ns*	*ns*	*ns*

Results as reported as mean±SD (standard deviation) of 3 independent replicates. A–B indicates significant differences (Tukey's test; $p<0.05$) between DP, wholegrain durum wheat pasta and EP; einkorn pasta samples. a–f indicates significant differences (Tukey's test; $p<0.05$) between samples. *ns*, not significant. DP, wholegrain durum wheat pasta EP; einkorn pasta. *$p< 0.05$, **$p< 0.01$, ***$p< 0.0001$. *ns*, not significant. C, cooking; D, digestion; T, type.

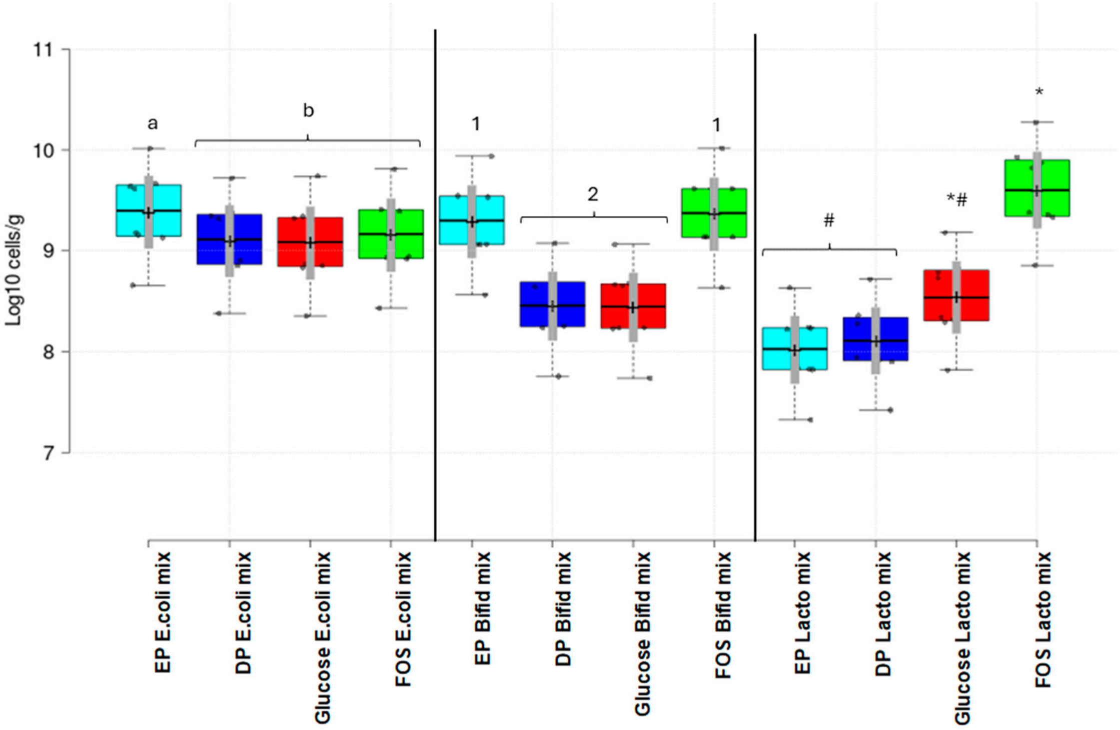

Figure S1. Selective bacterial growth on cooked pasta. Glucose = negative control; FOS (Fructooligosaccharides) = positive control. From left to right: *E. coli* mix = aerobic growth on BHI agar of *Escherichia coli* mix; Bifid = anaerobic growth on MRS cysteine agar of bifidobacteria mix; Lacto = Anaerobic growth on MRS agar of lactobacilli mix. DP, wholegrain durum wheat pasta EP; einkorn pasta. Letters, numbers and symbols indicate statistical differences by ANOVA and Tukey's *post hoc* test ($p<0.05$).

References

111. Masco, L.; Vanhoutte, T.; Temmerman, R.; Swings, J.; Huys, G. Evaluation of real-time PCR targeting the 16S rRNA and recA genes for the enumeration of *bifidobacteria* in probiotic products. *Int. J. Food Microbiol.* **2007**, *113*, 351-357. https://doi.org/10.1016/j.ijfoodmicro.2006.07.021.
112. Walter, J.; Tannock, G.W.; Tilsala-Timisjarvi, A.; Rodtong, S.; Loach, D.M.; Munro, K.; Alatossava, T. Detection and identification of gastrointestinal *Lactobacillus* species by using denaturing gradient gel electrophoresis and species-specific PCR primers. *Appl. Environ. Microbiol.* **2000**, *66*, 297-303. https://doi.org/10.1128/AEM.66.1.297-303.2000.
113. Malinen, E.; Kassinen, A.; Rinttila, T.; Palva, A. Comparison of real-time PCR with SYBR Green I or 5'-nuclease assays and dot-blot hybridization with rDNA-targeted oligonucleotide probes in quantification of selected faecal bacteria. *Microbiology (Reading)* **2003**, *149*, 269-277. https://doi.org/10.1099/mic.0.25975-0.
